# SNX16 activates c‐Myc signaling by inhibiting ubiquitin‐mediated proteasomal degradation of eEF1A2 in colorectal cancer development

**DOI:** 10.1002/1878-0261.12626

**Published:** 2020-01-10

**Authors:** Zhiyong Shen, Yongsheng Li, Yuan Fang, Mingdao Lin, Xiaochuang Feng, Zhenkang Li, Yizhi Zhan, Yuechen Liu, Tingyu Mou, Xiaoliang Lan, Yanan Wang, Guoxin Li, Jiping Wang, Haijun Deng

**Affiliations:** ^1^ Department of General Surgery Nanfang Hospital Southern Medical University Guangzhou China; ^2^ Department of Radiation Oncology Nanfang Hospital Southern Medical University Guangzhou China; ^3^ Department of Pathology Nanfang Hospital Southern Medical University Guangzhou China; ^4^ Division of Surgical Oncology Department of Surgery Brigham and Women's Hospital Harvard Medical School Boston MA USA

**Keywords:** cell proliferation, c‐Myc, colorectal cancer, eEF1A2, SNX16

## Abstract

Sorting nexin 16 (SNX16), a member of the sorting nexin family, has been implicated in tumor development. However, the function of SNX16 has not yet been investigated in colorectal cancer (CRC). Here, we showed that SNX16 expression was significantly upregulated in CRC tissues compared with normal counterparts. Upregulated mRNA levels of SNX16 predicted poor survival of CRC patients. Functional experiments showed that SNX16 could promote CRC cells growth both *in vitro* and *in vivo*. Knockdown of SNX16 induced cell cycle arrest and apoptosis, whereas ectopic overexpression of SNX16 had the opposite effects. Mechanistically, SNX16‐eukaryotic translation elongation factor 1A2 (eEF1A2) interaction could inhibit the degradation and ubiquitination of eEF1A2, followed by activation of downstream c‐Myc signaling. Our study unveiled that the SNX16/eEF1A2/c‐Myc signaling axis could promote colorectal tumorigenesis and SNX16 might potentially serve as a novel biomarker for the diagnosis and an intervention of CRC.

## Graphical abstract

Sorting nexin 16 (SNX16), which is significantly upregulated in colorectal cancer, could interact with and inhibit proteasome‐dependent ubiquitination of eukaryotic translation elongation factor 1 A2 (eEF1A2), thereby activating c‐myc signaling, Our study unveiled that SNX16/eEF1A2/c‐Myc signaling axis could promote colorectal tumorigenesis and SNX16 might potentially serve as a novel biomarker for the diagnosis and intervention of colorectal cancer.

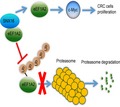

**Abbreviations**
CHX: cycloheximide
Co‐IP: co‐immunoprecipitation
CRC: colorectal cancer
eEF1A2: eukaryotic translation elongation factor 1 alpha 2
FDR: false discovery rate
GEO: Gene Expression Omnibus
GSEA: gene set enrichment analysis
IF: immunofluorescence
IHC: immunohistochemistry
N: normal colon mucosa
NES: normalized enrichment score
qRT‐PCR: quantitative real‐time PCR
SNX16: sorting nexin 16
SNXs: sorting nexins
T: human colon cancer tissue
TMA: tissue microarray

## 1 Introduction

Colorectal cancer (CRC) is the third most common cancer worldwide and was associated with 881 000 cancer‐related deaths in 2018 (Bray *et al.*, 2018). The initiation and development of CRC involves successive accumulation of genetic and epigenetic alterations that lead to a multistep and stepwise progression from normal mucosa to dysplasia and finally to carcinoma (Kuipers *et al.*, 2015; Luo *et al.*, 2013). Approximately eighty percent of CRC tumors can be distinguished by the accumulation of alterations in specific oncogenes (e.g., APC, KRAS, PIK3CA, SMAD4) and tumor suppressor genes, which thereby activate pathways that are critical for CRC tumorigenesis (Muller *et al.*, 2016). Aberrant activation of oncogenes is a key driver of uncontrolled cell proliferation in tumors (Nagarajan *et al.*, 2016). Thus, identification of novel oncogenes that are aberrantly overexpressed in CRC may provide insights into the oncogenic mechanisms underlying uncontrolled cell proliferation and lead to the discovery of new potential biomarkers for prognosis, diagnosis, and treatment of CRC patients.

Sorting nexins (SNXs) are a diverse group of proteins that contain the SNX‐PX domain and play a key role in membrane trafficking (Teasdale and Collins, 2012; Worby and Dixon, 2002). Some studies have shown that SNXs are also involved in the regulation of important signaling pathways associated with cancers. It is reported that SNX3 mediated intracellular cycling of the Wnt receptor Wntless (Harterink *et al.*, 2011). In addition, studies have shown that the expression levels of SNXs are closely related to the EGFR content in cells. In some tumors, high expression of SNXs can inhibit EGFR degradation (Chiow *et al.*, 2012; Liu *et al.*, 2006). Sorting nexin 16 (SNX16), a member of SNX family, is associated with vesicular K1 inflammatory virus infection, hepatitis C virus replication (Le Blanc *et al.*, 2005), and synaptic growth receptor transport (Blackham *et al.*, 2010). SNX16 has been implicated in the development of various tumors. SNX16 was found to be overexpressed in the blood cells of bladder cancer patients (Osman, 2006). In addition, SNX16 levels were significantly higher in ovarian cancer tissue than in normal tissues (Pharoah *et al.*, 2013). Previous studies have also suggested that SNX16 exhibited alternative splicing in certain melanoma cell lines and could interact with 32 SNPs that are known risk factors for prostate cancer (Tao *et al.*, 2012; Watahiki *et al.*, 2004). However, the expression and biological function of SNX16 with regard to CRC has not been thoroughly investigated.

c‐Myc is a well‐established cancer driver gene that plays essential roles in multiple biological processes including cell proliferation, cell growth, apoptosis, and energy metabolism (Dang, 2012; Gong *et al.*, 2018). Constitutive upregulation of c‐Myc is believed to be the basis of a variety of tumors, including CRC (Gong *et al.*, 2018). However, to the best of our knowledge, the mechanism by which SNX16 regulates c‐Myc signaling to control of cell proliferation in CRC has never been reported.

Here, for the first time, we evaluated the expression pattern and clinical significance of SNX16 in CRC, aiming to elucidate the functions and molecular mechanisms of SNX16 both *in vitro* and *in vivo*. Our study provides new mechanistic insights into the crucial roles of SNX16 in the activation of c‐Myc signaling *via* inhibition of eukaryotic translation elongation factor 1A2 (eEF1A2) ubiquitination, providing a potential marker and novel intervention targets for CRC.

## 2 Materials and methods

### 2.1 Patient samples and cell culture

This study was approved by the Institutional Research Medical Ethics Committee of Nanfang Hospital. The experiments were undertaken with the understanding and written consent of each participant, which was in accordance with the Declaration of Helsinki. All human CRC tissue samples were collected from the Department of General Surgery, Nanfang Hospital, Southern Medical University. Twenty pairs of CRC specimens (CRC and adjacent nontumor tissues) were used for quantitative real‐time PCR (qRT‐PCR). Nine pairs of CRC specimens were used for western blot analyses. Fifteen paired CRC and adjacent normal tissues were used for immunohistochemical (IHC) analysis. A tissue microarray (TMA), involving a total of 193 CRC patients who underwent colorectal resections from November 2013 to June 2014 in Nanfang Hospital, Southern Medical University (Guangzhou, China), was used to analyze the correlations among SNX16, eEF1A2, and c‐Myc expression.

The datasets used were downloaded from the public Gene Expression Omnibus (GEO) and Oncomine (http://www.oncomine.org) databases. We evaluated the correlation of SNX16 expression levels with patient survival in CRC using the R2: Genomics Analysis and Visualization Platform (a biologist friendly Web‐based genomics analysis and visualization application; http://r2.amc.nl). CRC cell lines (SW1116, HT29, 174T, CaCO2, HCT115, DLD1, SW480, RKO, SW620, LoVo, HCT116) were obtained from the American Type Culture Collection (Manassas, VA, USA).

### 2.2 Western blot analysis and quantitative real‐time PCR

Proteins were separated on SDS/PAGE gels and transfer to polyvinylidene fluoride membranes. The membranes were incubated with different primary antibody (Table S2) in TBS‐Tween 20 at 4 °C overnight. Following incubation with the appropriate secondary antibody, the membranes were visualized using the Luminata Chemiluminescent Detection Kit (Millipore, Burlington, MA, USA). Total RNA extraction and qRT‐PCR were performed, as previously described (Shen *et al.*, 2019b).

### 2.3 Immunohistochemical analysis

Human CRC tissue samples and mice subcutaneous tumor samples were fixed in 4% paraformaldehyde for 24 h and were embedded in paraffin; 5‐μm‐thick sections were prepared for IHC staining. Deparaffinized sections were quenched to eliminate endogenous peroxidase activity, followed by antigen retrieval and blocking procedures. Then, the slices were incubated with different primary antibodies at 4°C overnight. Subsequently, the slices were incubated with biotinylated secondary antibody and visualized using a DAB kit.

To evaluate the results, two individuals scored all the sections independently. The final IHC scores were generated by combining the scores for the proportion of positive tumor cells and the intensity of staining (Ni *et al.*, 2017). The proportion score was determined as follows: 0, no positive tumor cells; 1, 1–25%; 2, 26–50%; 3, 51–75%; 4, > 75%. The intensity of staining was evaluated as follows: 0, no staining; 1, weak staining; 2, intermediate staining; 3, strong staining. The comprehensive IHC score was calculated by multiplying the proportion score by the staining intensity score. When the SNX16 expression score was higher than the average score, the SNX16 expression in these CRC samples was defined as high; otherwise, it was defined as low.

### 2.4 Cell transfection

An optimized SNX16‐knockdown lentivirus expressing LV‐SNX16‐RNAi (sh‐SNX16; GeneChem, Shanghai, China) was used to transfect HT29 and LoVo cells. Cells were transfected with empty lentivirus as a negative control (sh‐NC). The SNX16‐overexpressing cell line was constructed using the SNX16‐overexpressed vectors (LV‐SNX16), and a NC (LV‐NC) cell line was also generated. Transfection procedures were performed according to the manufacturer's instructions.

### 2.5 Cell proliferation assay and colony formation assay

Cell growth was determined using the MTT assay (Sigma, St. Louis, MO, USA) according to the manufacturer's instructions. A total of 1 × 10^3^ transfected cells per well were seeded into 96‐well plates, and cell viability was assessed every 24 h following the manufacturer's protocol. EdU assays were used to examine the effect of SNX16 on DNA replication. The cells were treated with to 50 μm of 5‐ethynyl‐2'‐deoxyuridine (Ribobio, Guangzhou, China) for 2 h and processed according to the manufacturer's instruction. Then, the DNA contents of the cells were stained with Hoechst33342 and visualized by a fluorescence microscope. For the colony formation assay, transfected cells were seeded into six‐well plates. At the end of experiments, colonies were fixed with methanol and stained with 0.1% crystal violet (Leagene, Beijing, China).

### 2.6 Flow cytometry

Transfected cells were harvested after 5‐Fu（40 μg·mL^−1^) treatment for 48 h. Cells were collected and apoptosis was detected by using the Annexin V‐APC/PI Apoptosis Detection Kit (KeyGen Biotech, Nanjing, China). For cell cycle analysis, cells were incubated with RNase and PI staining using the Cell Cycle Detection Kit. The detailed procedures were performed according to instructions provided (KeyGen Biotech).

### 2.7 Animal models

To evaluate *in vivo* tumorigenesis, 5 × 10^6^ transfection cells were subcutaneously injected into the left or right flanks of 4‐week‐old nude mice (five mice in each group). The tumor size was measured every 3 days, and the tumor volume was calculated as (length × width^2^)/2 (Shen *et al.*, 2019b). All nude mice were obtained from Laboratory Animal Center of Southern Medical University. All animal experiments were approved by the Animal Care and Use Committee of Southern Medical University.

### 2.8 Co‐immunoprecipitation and mass spectrometry

The total protein from HT29 cells was extracted in radioimmunoprecipitation assay buffer supplemented with proteinase/phosphatase inhibitors. Total cell extracts were incubated with anti‐SNX16 (Santa Cruz, MA, USA) and IgG (as a negative control) with gentle shaking overnight at 4 °C, followed by the addition of protein A/G‐agarose beads (Thermo Scientific, Waltham, MA, USA) for an additional 4 h. The beads were washed and resuspended in PBS and 5× loading buffer and boiled for 5 min. The proteins were separated by SDS/PAGE, followed by silver staining. Candidate bands were subjected to mass spectrometric analysis for protein identification.

### 2.9 Immunofluorescence staining

Cells were fixed in a 4% paraformaldehyde solution, and 0.5% Triton solution was added to disrupt the cytomembrane. After blocking in 1% BSA at room temperature for 20 min, the cells were incubated with primary antibodies of interest at 4 °C overnight. After washing with PBS three times, a fluorescent secondary antibody and 4',6‐diamidino‐2‐phenylindole staining kit were used in the dark to detect the bound primary antibody and cell nuclei, respectively. The cells were observed under an inverted microscope.

### 2.10 Ubiquitination assay

SNX16‐knockdown HT29 cells or SNX16‐overexpressing SW480 cells and control cells were treated with 30 µm MG132 for 12 h to block proteasomal degradation. The lysates were immunoprecipitated with anti‐eEF1A2 (Proteintech, Rosemont, IL, USA) antibodies or anti‐IgG on protein A/G beads (Thermo Scientific) overnight at 4 °C with rotation and then boiled in SDS buffer. Eluates were subjected to western blotting using anti‐K48‐linked poly‐ubiquitination antibody to evaluate the proteasome‐dependent ubiquitination level.

### 2.11 Statistical analysis

Statistical analysis was carried out with spss 22.0 (SPSS Inc., Chicago, IL, USA) statistical software package or graphpad prism 7.0 software (GraphPad Software Inc., San Diego, CA, USA). *T*‐tests were used to evaluate differences between two groups of variables. Survival curves were obtained by the Kaplan–Meier survival analysis. Cox proportional hazards regression was used to identify independent factors that have a significant impact on patient survival. Correlations among SNX16 expression, eEF1A2 expression, and c‐Myc expression in the TMA were analyzed with Spearman's rank correlation. Probability values from the two‐tailed test that were < 0.05 were considered significant.

## 3 Results

### 3.1 SNX16 is overexpressed in colorectal cancer and is correlated with poor prognosis

**Figure 1:**
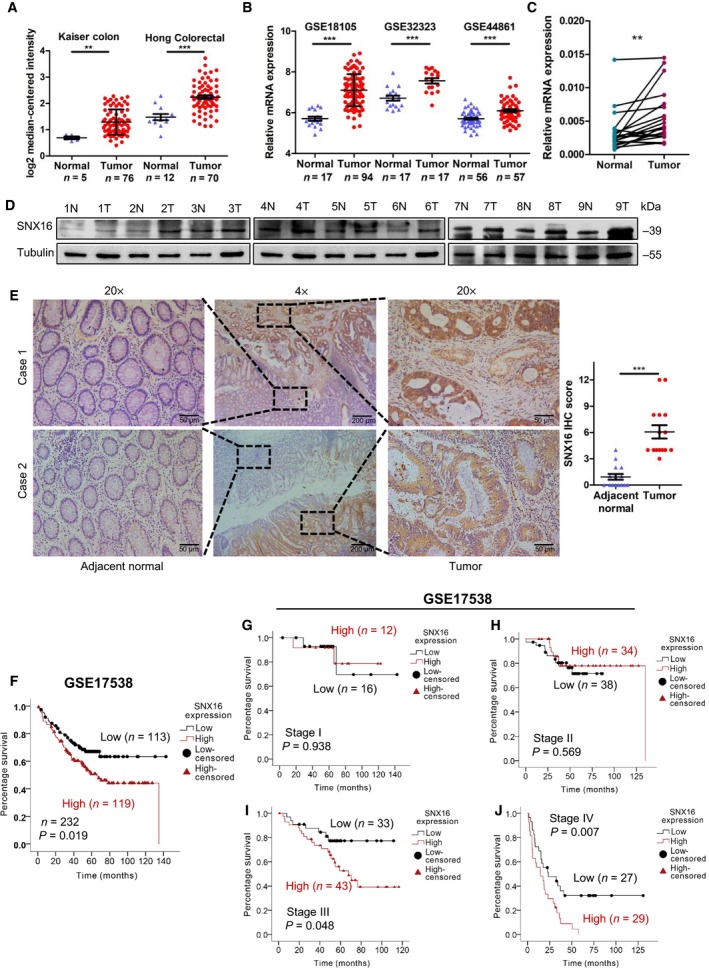
SNX16 is overexpressed in CRC, and overexpression of SNX16 is associated with poor prognosis. (A, B) SNX16 mRNA expression was analyzed using data from the Kaiser colon cohort, Hong colorectal cohort and GEO dataset (http://www.ncbi.nlm.nih.gov/geo/query/acc.cgi?acc=GSE18105, http://www.ncbi.nlm.nih.gov/geo/query/acc.cgi?acc=GSE32323, and http://www.ncbi.nlm.nih.gov/geo/query/acc.cgi?acc=GSE44861). **P < 0.01, ***P < 0.001. (C) qRT‐PCR analysis of SNX16 mRNA expression in 20 paired human CRC tissues. SNX16 levels were quantified relative to matched adjacent nontumor tissues and were normalized to GAPDH levels. The data are presented as the mean ± SEMs of three independent experiments. Student's *t*‐test was performed. **P < 0.01. (D) Western blot analysis of SNX16 protein expression in nine paired CRC tissues and adjacent normal tissues. Tubulin was used as an internal control. (E) IHC analysis of the SNX16 protein in CRC tissues and adjacent normal intestinal epithelium tissues. Representative images of the staining are shown. ***P < 0.001. Scale bar, 200 μm (4×), 50 μm (20×). (F) Kaplan–Meier analysis of the overall survival of CRC patients based on the expression level of SNX16 mRNA in the GEO dataset http://www.ncbi.nlm.nih.gov/geo/query/acc.cgi?acc=GSE17538 (six patients were not included in the analysis due to a lack of follow‐up data). (G–J) Kaplan–Meier analysis of overall survival of patients in stages I, II, III, and IV after stratification by TNM stage.

To explore the expression of SNX16 in CRC, we first analyzed SNX16 expression levels in normal and CRC tissues by bioinformatics analysis. Microarray data from Oncomine and the public GEO database (http://www.ncbi.nlm.nih.gov/geo/query/acc.cgi?acc=GSE18105, http://www.ncbi.nlm.nih.gov/geo/query/acc.cgi?acc=GSE32323, and http://www.ncbi.nlm.nih.gov/geo/query/acc.cgi?acc=GSE44861) revealed that SNX16 was significantly upregulated in tumors compared to normal tissues (Fig. 1A,B; Fig. S1). Consistent with the publicly available data, qRT‐PCR (*n* = 20), western blot analysis (*n* = 9), and IHC analysis (*n* = 15) showed that the mRNA and protein levels of SNX16 were similarly elevated in CRC tissues compared to the corresponding adjacent normal mucosa (Fig. 1C–E).

Finally, we investigated the clinical relevance of SNX16 by using public datasets. The results of the Kaplan–Meier survival analysis suggested that patients with high SNX16 expression levels (*P* = 0.019) had adverse clinical outcomes among 232 CRC patients from the GEO database (http://www.ncbi.nlm.nih.gov/geo/query/acc.cgi?acc=GSE17538; Fig. 1F), especially in stage III (*P* = 0.048) or IV (*P* = 0.007) patients with CRC but not in stage I (*P* = 0.938) or II (*P* = 0.569) patients (Fig. 1G–J). Univariable Cox regression model analysis revealed that poor differentiation (hazard ratio, HR: 1.52; 95% confidence interval, 95% CI: 1.168–1.966; *P* = 0.002), advanced clinical stage (HR: 2.73; 95% CI: 2.087–3.558; *P* = 0.000), and high SNX16 expression (HR: 1.64; 95% CI: 1.079–2.500; *P* = 0.021) were significantly associated with poor survival. Furthermore, multivariable Cox regression analysis revealed that high SNX16 expression is an independent prognostic factor for poor survival (HR: 1.75, 95% CI: 1.113–2.737; *P* = 0.015; Table 1). Additionally, we analyzed the prognostic value of SNX16 for CRC patients using the R2: Genomics Analysis and Visualization Platform (http://r2.amc.nl). High expression of SNX16 was associated with poor overall survival and relapse‐free survival time (Fig. S2).

**Table 1:** Univariable and multivariable Cox regression analyses of clinical characteristics associated with prognosis of 232 CRC patients (http://www.ncbi.nlm.nih.gov/geo/query/acc.cgi?acc=GSE17538).

.Variable	All case^a^	Univariable analysis^b^		Multivariable analysis^b^	
		HR (95% CI)	*P*‐value	HR (95% CI)	*P*‐value
Gender					
Male	122	1.035 (0.689–1.555)	0.869	–	–
Female	110				
Age (years)					
< 65	110	1.062 (0.707–1.596)	0.772	–	–
≥ 65	122				
Differentiation^c^					
Well/moderate	183	1.515 (1.168–1.966)	0.002	1.355 (1.037–1.771)	0.026
Poor	30				
TNM stage					
I	28	2.725 (2.087–3.558)	0.000	2.742 (2.061–3.646)	0.000
II	72				
III	76				
IV	56				
SNX16 expression					
Low expression	113	1.642 (1.079–2.500)	0.021	1.745 (1.113–2.737)	0.015
High expression	119				

a Six cases missing follow‐up data

b Cox regression model (method = Enter)

c Nineteen cases missing Differentiation data.

### 3.2 SNX16 promotes colorectal cancer cells proliferation *in vitro*

**Figure 2:**
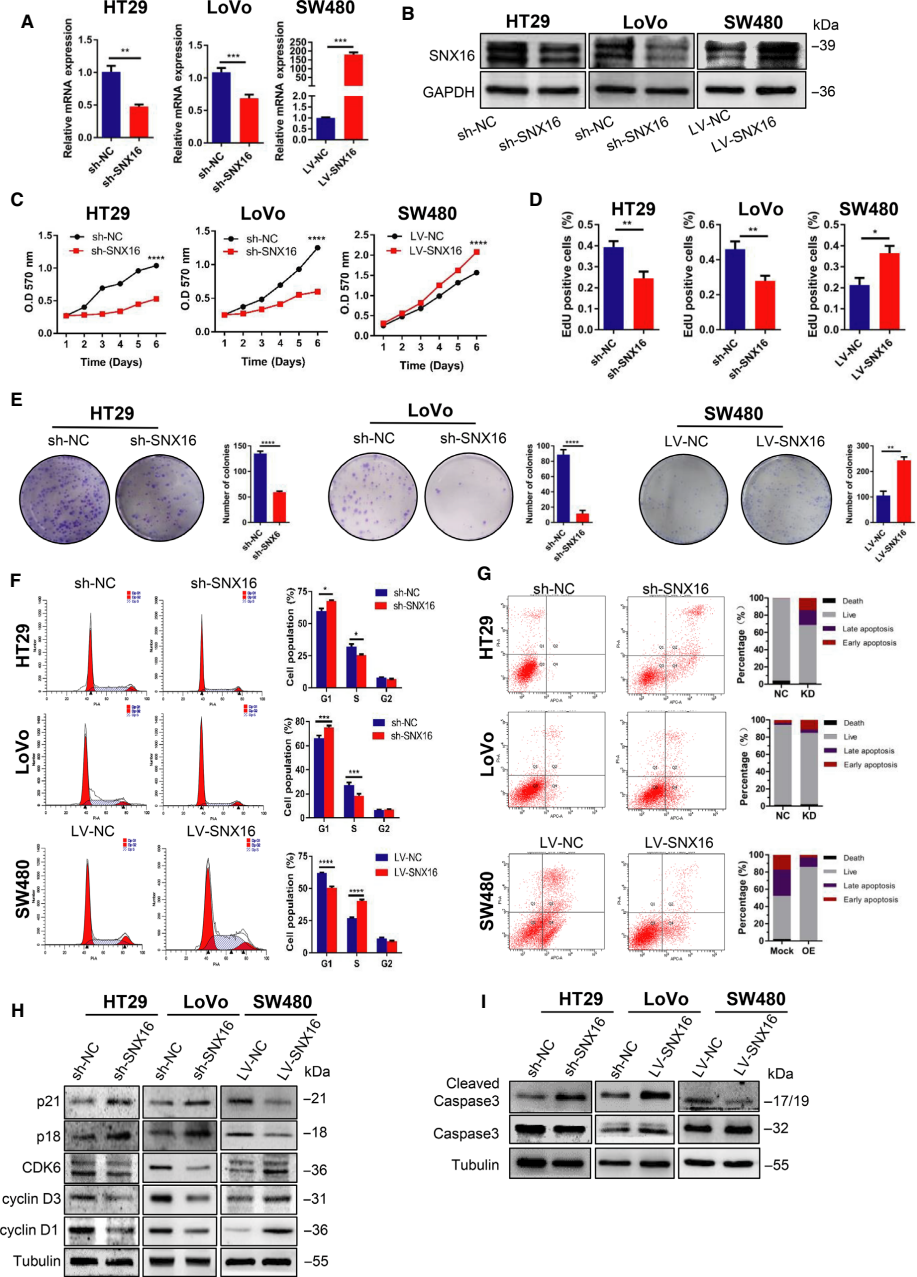
SNX16 promotes CRC cells growth *in vitro.* (A, B) Effects of SNX16 knockdown and overexpression were analyzed by qRT‐PCR and western blot analysis. GAPDH was used as the loading control. **P < 0.01, ***P < 0.001. (C). Knockdown of SNX16 inhibited HT29 and LoVo cell proliferation, whereas ectopic expression of SNX16 promoted SW480 cell proliferation, as determined by the MTT assay. The results are shown as the means ± SEMs (*n* = 5), ****P < 0.0001. (D) The effect of knockdown or overexpression of SNX16 on CRC cell proliferation capacity was analyzed by the EdU assay. The results are shown as the means ± SEMs (*n* = 5), *P < 0.05, **P < 0.01. (E) Effect of SNX16 on colony formation by CRC cells. The results are shown as means ± SEMs (*n* = 3), **P < 0.01, ****P < 0.0001. (F) SNX16 accelerated the G1‐S transition of the cell cycle, as indicated by flow cytometry. *P < 0.05, **P < 0.01, ***P < 0.001, ****P < 0.0001. (G) SNX16 inhibited cell apoptosis, as determined by Annexin V‐APC/PI staining and flow cytometry. (H) The effect of knockdown or overexpression of SNX16 on cell cycle regulators, including p21, p18, CDK6, cyclin D1, and cyclin D3, was determined by western blotting. (I) The effect of knockdown or overexpression of SNX16 on apoptosis‐associated protein caspase‐3 was analyzed by western blotting. Tubulin was used as the loading control.

Given previous results, we further evaluated the functional role of SNX16 in CRC cells. We first analyzed the endogenous levels of SNX16 expression in 11 CRC cell lines by qRT‐PCR and western blotting (Fig. S3). HT29 and LoVo cells with relatively high endogenous SNX16 expression were selected to establish stable SNX16 knockdown cell lines, while SW480 cells with low endogenous SNX16 expression were selected to establish stable SNX16‐overexpressing cell lines (Fig. 2A,B). As evidenced by MTT assays, EdU assays and colony formation assays, knockdown of SNX16 expression in HT29 and LoVo cells significantly inhibited cell growth. Conversely, ectopic expression of SNX16 in SW480 cells promoted cell proliferation and viability (Fig. 2C–E; Fig. S4). However, transwell assays showed that the downregulation or upregulation of SNX16 had no significant effect on the migration capacity of CRC cells compared with control cells (Fig. S5).

### 3.3 Knockdown of SNX16 induces cell cycle arrest and apoptosis in colorectal cancer cells

To investigate the mechanism underlying decreased cell proliferation in SNX16‐knockdown HT29 and LoVo cells, the effect of SNX16 knockdown on cell cycle progression was analyzed by flow cytometry. Our results revealed that knockdown of SNX16 in HT29 and LoVo cells significantly decreased the S‐phase cell population but increased the G0 to G1 phase cell population (Fig. 2F). In contrast, ectopic expression of SNX16 in SW480 cells significantly decreased the cell population in the G0 to G1 phase but increased the S‐phase cell population. Furthermore, we analyzed kinetic of cell cycle progression after releasing cancer cells from G1 phase synchronously and obtained similar results (Fig. S5).

To further explore the underlying molecular mechanisms that promote G1 to S transition mediated by SNX16, the expression of some of the cell cycle regulators was determined. Western blot analysis showed that knockdown of SNX16 expression enhanced the expression of G1 gatekeepers such as p21 and p18 and reduced the expression of cyclin D1, cyclin D3, and CDK6 (Fig. 2H). However, overexpression of SNX16 in SW480 cells had the opposite effects on the expression of the above mentioned key cell cycle regulators.

Analysis of apoptosis by flow cytometry revealed that SNX16 knockdown induced a significant increase in the total apoptosis rate in HT29 and LoVo cells (Fig. 2G). In contrast, ectopic SNX16 expression markedly decreased the total apoptosis rate in SW480 cells (Fig. 2G). Subsequently, we determined the expression levels of caspase‐3, which plays a key role in apoptosis. Western blot analysis showed that the expression of cleaved forms of caspase‐3 was upregulated in stable SNX16‐knockdown HT29 and LoVo cells (Fig. 2I). Conversely, ectopic expression of SNX16 in SW480 cells led to decreased expression of cleaved forms of caspase‐3 (Fig. 2I). Taken together, our results indicated that SNX16 exerts its oncogenic effect by inhibiting apoptosis and promoting the cell cycle progression of CRC cells.

### 3.4 SNX16 promotes colorectal cancer cell proliferation by activating the c‐Myc signaling pathway

**Figure 3:**
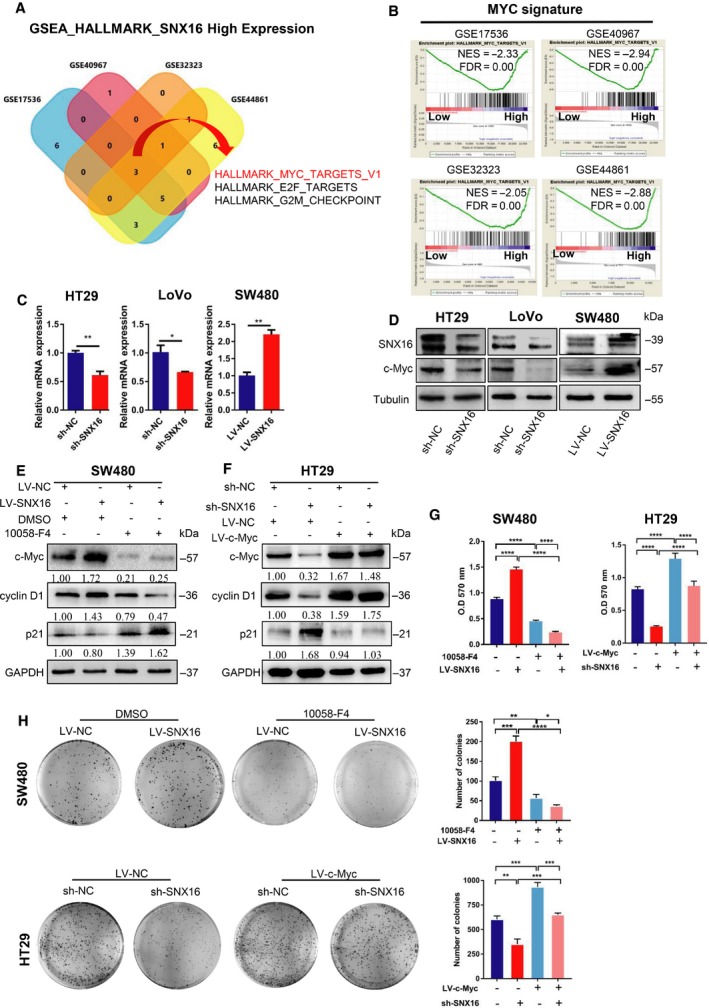
SNX16 promotes CRC cell proliferation by activating the c‐Myc signaling pathway. (A, B) GSEA demonstrated that MYC was positively associated with a high‐SNX16‐expression CRC group (http://www.ncbi.nlm.nih.gov/geo/query/acc.cgi?acc=GSE17536, http://www.ncbi.nlm.nih.gov/geo/query/acc.cgi?acc=GSE40967, http://www.ncbi.nlm.nih.gov/geo/query/acc.cgi?acc=GSE32323, and http://www.ncbi.nlm.nih.gov/geo/query/acc.cgi?acc=GSE44861). NES, normalized enrichment score; FDR, false discovery rate. (C, D) The expression of SNX16 and c‐Myc in SNX16‐knockdown or SNX16‐overexpressing cells was measured by qRT‐PCR and western blotting. (E) Western blots of the indicated proteins in SW480 cells (LV‐NC vs. LV‐SNX16) treated with the c‐Myc inhibitor 10058‐F4. (F) Western blots of indicated proteins in HT29 cells (sh‐NC vs. sh‐SNX16) with/without ectopic expression of c‐Myc. (G) MTT assay. The results are shown as the means ± SEMs (*n* = 5), ****P < 0.0001. (H) Colony formation assays. The results are shown as the means ± SEMs (*n* = 3), *P < 0.05, **P < 0.01, ***P < 0.001, ****P < 0.0001.

To explore the downstream role of SNX16 in CRC proliferation, we performed gene set enrichment analysis (GSEA) on the microarray data from http://www.ncbi.nlm.nih.gov/geo/query/acc.cgi?acc=GSE17536, http://www.ncbi.nlm.nih.gov/geo/query/acc.cgi?acc=GSE40967, http://www.ncbi.nlm.nih.gov/geo/query/acc.cgi?acc=GSE32323, and http://www.ncbi.nlm.nih.gov/geo/query/acc.cgi?acc=GSE44861. The results revealed that MYC was positively associated with high SNX16 expression in the CRC group (Fig. 3A,B). Moreover, the results of western blot and qRT‐PCR analysis suggested that knockdown of SNX16 in HT29 and LoVo cells reduced the expression of c‐Myc, whereas overexpression of SNX16 in SW480 cells increased the expression of c‐Myc (Fig. 3C,D). Previous studies have reported that c‐Myc is a powerful oncogene involved in the regulation of cell proliferation, apoptosis, differentiation, and other biological processes (Dang, 2012; Sheikh Zeineddini *et al.*, 2019; Shen *et al.*, 2019a; Zhang *et al.*, 2019). Therefore, we further investigated whether the pro‐proliferative effects of SNX16 are dependent on c‐Myc signaling. First, we treated SNX16‐overexpressing SW480 cells with a c‐Myc inhibitor (10058‐F4) and found that SNX16‐overexpressing SW480 cells were more sensitive to the inhibitors than control cells; this increase in sensitivity was characterized by a more dramatic change in the expression of downstream cyclin D1 protein downregulation and p21 protein upregulation (Fig. 3E). Next, we constructed stable SNX16‐knockdown and c‐Myc‐overexpressing HT29 cells. We found that ectopic expression of c‐Myc significantly increased downstream cyclin D1 in HT29 cells with/without SNX16 knockdown, and decreased downstream p21 in HT29 cells with SNX16 knockdown (Fig. 3F). In addition, overexpression of c‐Myc abrogated the SNX16 knockdown‐mediated repression of CRC cell proliferation, whereas the inhibition of c‐Myc expression significantly inhibited the proliferation of SNX16 overexpressing CRC cells compared to that of control cells. (Fig. 3G,H; Fig. S7). Taken together, these observations confirmed that SNX16 promotes the proliferation of CRC cells by activation of the c‐Myc signaling pathway.

### 3.5 The oncoprotein eEF1A2 is an interactive factor of SNX16

**Figure 4:**
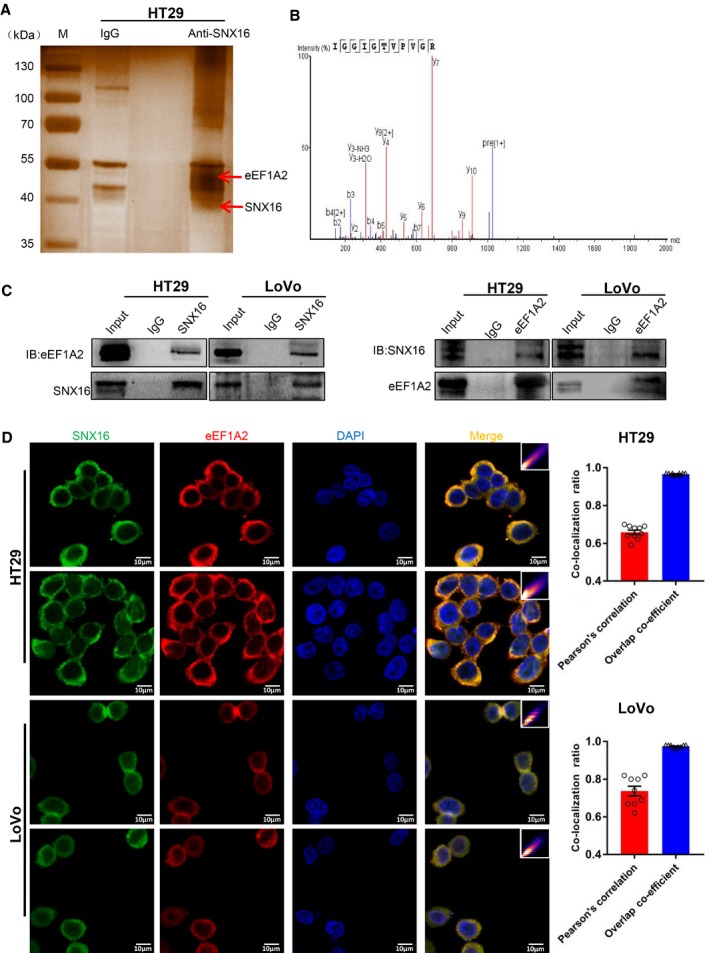
SNX16 interacts with eEF1A2 in CRC. (A, B). The proteins pulled down by using anti‐SNX16 and IgG were visualized by silver staining. Co‐IP of SNX16‐binding proteins followed by mass spectrometry led to the identification eEF1A2 as a SNX16‐binding protein. (C) The protein–protein interactions between SNX16 and eEF1A2 were confirmed by Co‐IP in HT29 and LoVo cells. (D) IF analyses of colocalization of SNX16 (green) and eEF1A2 (red) in HT29 and LoVo cells (left). The quantitative values of the colocalization of SNX16 and eEF1A2 (right). The scale bars represent 10 µm.

Using co‐immunoprecipitation (Co‐IP) experiments, we found that SNX16 could not interact with c‐Myc (Fig. S8). Considering this inability, how does SNX16 activate the c‐Myc signaling pathway? To gain insight into the molecular mechanisms by which SNX16 regulates c‐Myc signaling pathway in CRC, we performed Co‐IP with an anti‐SNX16 antibody to pull down potential interacting proteins from HT29 cells, followed by mass spectrometric analysis for protein identification (Fig. 4A,B). eEF1A2 was one of the most abundant proteins identified by mass spectrometry (Fig. 4B), suggesting a potential interaction between eEF1A2 and SNX16.

Human eEF1A is a member of the G protein family and one of four subunits that constitute eukaryotic elongation factor 1 (Browne and Proud, 2002; Ejiri, 2002). eEF1A2, an isoform of the eEF1A protein, is aberrantly upregulated in many tumor tissues (Cao *et al.*, 2009; Kulkarni *et al.*, 2007; Pinke *et al.*, 2008; Scaggiante *et al.*, 2012) and has been identified as a tumor‐associated protein (Lee and Surh, 2009; Pellegrino *et al.*, 2014). eEF1A2 has been reported to be involved in protein translation, the cell cycle, and apoptosis (Chang and Wang, 2007; Lee *et al.*, 2013). Therefore, we suspect that the role of SNX16 in the inhibition of apoptosis and promotion of cell cycle progression is mediated by eEF1A2. Furthermore, Co‐IP experiments using anti‐SNX16 antibodies in HT29 and LoVo cells confirmed that SNX16 binds to eEF1A2, and reciprocal Co‐IP further confirmed the interaction between them by using anti‐eEF1A2 antibody to coprecipitate SNX16 in these cells (Fig. 4C). Immunofluorescence (IF) analyses showed that SNX16 and eEF1A2 were colocalized in the cytoplasm of HT29 and LoVo cells (Fig. 4D). Thus, we identified eEF1A2 as an interaction partner of SNX16.

### 3.6 SNX16 stabilizes the oncoprotein eEF1A2 by inhibiting ubiquitination

**Figure 5:**
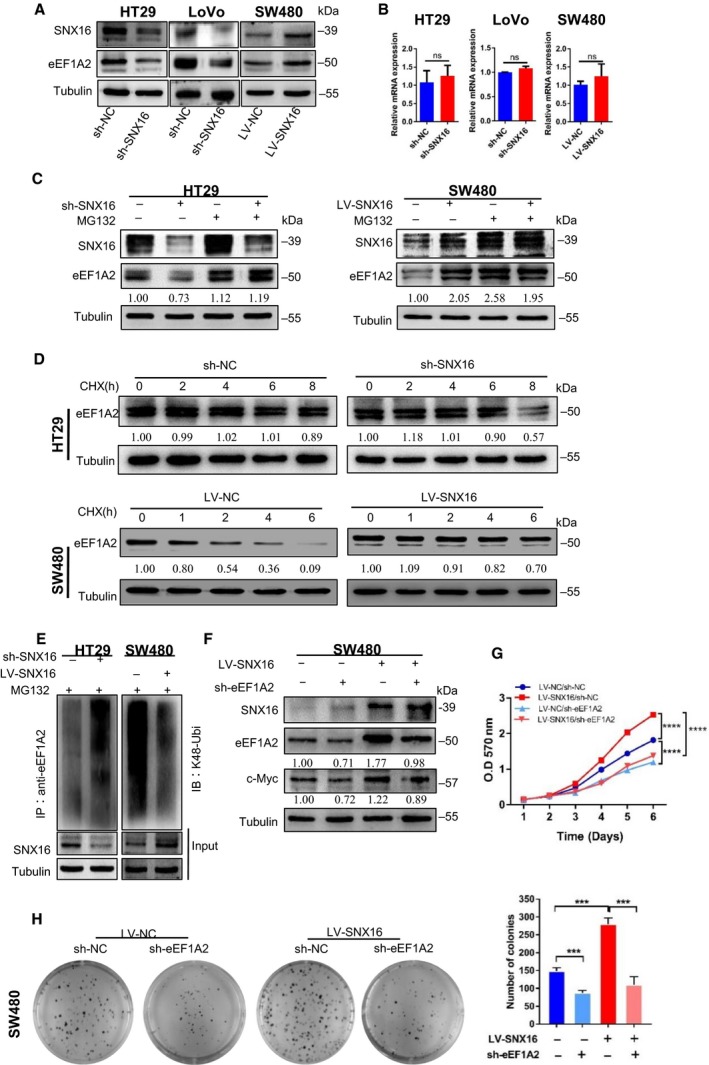
SNX16 activates the c‐Myc signaling pathway by inhibiting eEF1A2 degradation in CRC. (A) The expression of SNX16 and eEF1A2 in SNX16‐knockdown or SNX16‐overexpressing cells was measured by western blotting. Tubulin was used as the loading control. (B) The expression of SNX16 and eEF1A2 in SNX16‐knockdown or SNX16‐overexpressing cells was measured by qRT‐PCR. GAPDH was used as the loading control. (C) eEF1A2 levels were determined in SNX16‐knockdown HT29 cells and SNX16‐overexpressing SW480 cells before and after MG132‐mediated stimulation by western blotting. (D) SNX16‐knockdown HT29 cells and SNX16‐overexpressing SW480 cells were exposed to CHX (20 μg·mL^−1^) at the indicated time point, and degradation of eEF1A2 was detected by western blot analysis. (E) SNX16‐knockdown HT29 cells and SNX16‐overexpressing SW480 cells were treated with MG132, and the level of ubiquitin‐bound eEF1A2 was then measured. (F) Upregulation of c‐Myc induced by SNX16 was attenuated upon knockdown of eEF1A2 in SNX16‐overexpressing cells. (G) MTT assay. The results are shown as the means ± SEMs (*n* = 5), ****P < 0.0001. (H) Colony formation assays. Results are shown as mean ± SEM (*n* = 3), *P < 0.05, ***P < 0.001

Subsequently, we evaluated the impact of SNX16 on eEF1A2 expression. The results of western blot analyses suggested that knockdown of SNX16 in HT29 and LoVo cells decreased the expression of eEF1A2, whereas ectopic expression of SNX16 in SW480 cells induced the upregulation of eEF1A2 (Fig. 5A). However, the mRNA levels of eEF1A2 were not significantly altered in cells with either SNX16 knockdown or overexpression (Fig. 5B), suggesting that SNX16 might positively regulate eEF1A2 expression at the posttranscriptional level. Previous research has shown that eEF1A2 can be degraded *via* the ubiquitin–proteasome pathway (Sanges *et al.*, 2012). Therefore, we hypothesized that the interaction between SNX16 and eEF1A2 might stabilize eEF1A2 by inhibiting the ubiquitin‐mediated degradation of this protein. To confirm this hypothesis, we evaluated the effects of changes in SNX16 expression on eEF1A2 protein levels, either with or without the proteasome inhibitor MG132 (Wang *et al.*, 2019). Western blot analysis showed that SNX16 no longer affected eEF1A2 expression in HT29 and SW480 cells after treatment with MG132 (Fig. 5C). Then, we further pretreated the cells with cycloheximide (CHX) (Meng *et al.*, 2018) to block protein synthesis and to determine the stability of eEF1A2. The results indicated that the half‐life of eEF1A2 expression was significantly reduced in SNX16‐knockdown HT29 cells and elevated in SNX16‐overexpressing SW480 cells compared with that in parental cells within the same time interval (Fig. 5D). The results of the *in vitro* ubiquitination assay showed a significant increase in the level of ubiquitinated eEF1A2 protein in SNX16‐knockdown cells. However, overexpression of SNX16 reduced eEF1A2 ubiquitination (Fig. 5E). Taken together, these results indicated that SNX16 stabilized the expression of the oncoprotein eEF1A2 by regulating eEF1A2 ubiquitination in CRC cells.

### 3.7 SNX16 activates the c‐Myc signaling pathway by inhibiting eEF1A2 degradation

We then examined the functional role of eEF1A2 in CRC and found that knockdown of eEF1A2 significantly inhibited growth (Fig. S9B) and colony formation ability (Fig. S9C) of HT29 cells. Moreover, SNX16 expression was unchanged following knockdown of eEF1A2 in HT29 cells (Fig. S9A), suggesting that eEF1A2 was a downstream effector of SNX16. To determine whether SNX16 regulated c‐Myc expression *via* the eEF1A2 protein, we constructed stable SNX16‐overexpressing and eEF1A2‐knockdown SW480 cells. We found that knockdown of eEF1A2 expression blocked the effect of SNX16 on c‐Myc expression, which suggested that eEF1A2 was a key factor for SNX16‐mediated activation of the c‐Myc signaling pathway (Fig. 5F). In addition, functional rescue experiments showed that the proliferative effect of SNX16 on CRC cells was reversed after knockdown of eEF1A2 (Fig. 5G,H). Thus, our results suggested that eEF1A2 is indispensable for SNX16‐mediated tumor‐promoting functions in CRC cells. Collectively, our data indicated that SNX16 activates the c‐Myc signaling pathway by inhibiting eEF1A2 degradation.

### 3.8 SNX16 promotes subcutaneous xenograft tumor growth in nude mice

**Figure 6:**
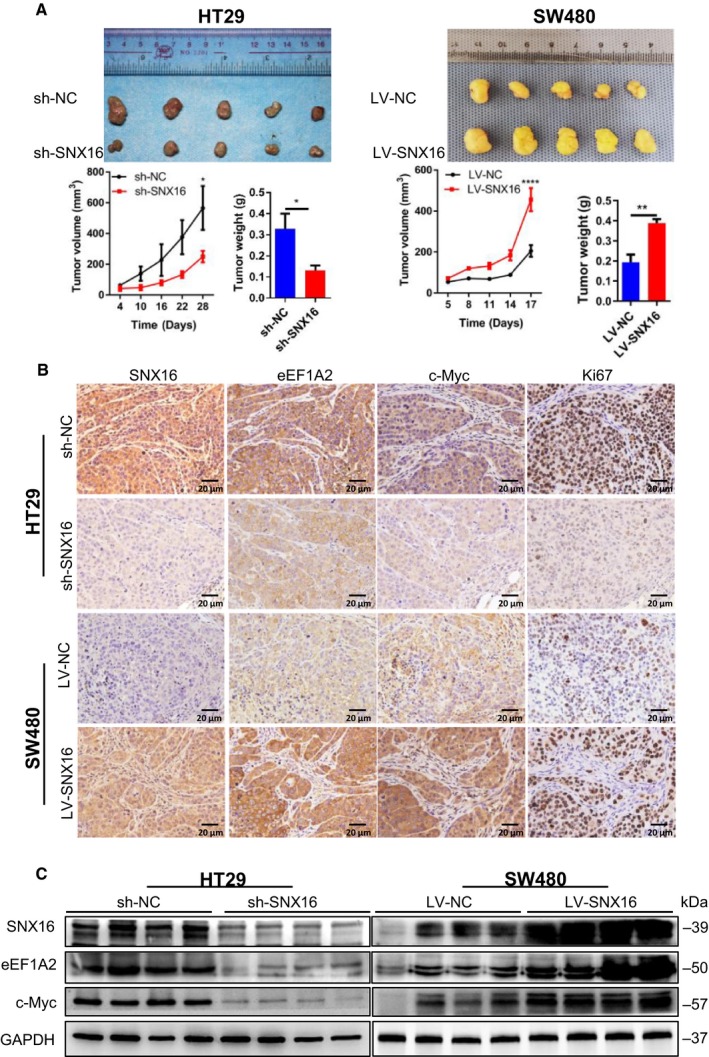
SNX16 promotes tumorigenesis in mice. (A) Subcutaneous xenograft tumor growth in nude mice was measured and compared in HT29 (sh‐NC vs. sh‐SNX16) and SW480 (LV‐NC vs. LV‐SNX16) cell lines. Results are shown as mean ± SEM, *P < 0.05, **P  < 0.01, ****P < 0.0001. (B) Representative images of IHC staining of SNX16, eEF1A2, c‐Myc, and Ki67 on tumor sections. Scale bar, 20 μm (40×). (C) The expression levels of the indicated proteins in these tumors were examined by western blotting. GAPDH was used as the loading control.

Based on our *in vitro* findings, we detected the functions of SNX16 *in vivo* using subcutaneous xenograft models. We performed a subcutaneous xenograft assay in nude mice using stable SNX16‐knockdown CRC cells or stable SNX16‐overexpressing CRC cells or empty vector. The results showed that knockdown of SNX16 in HT29 cells significantly suppressed tumor growth by 57% and lowered the tumor weight by 60% compared to the negative controls (Fig. 6A). Reversely, overexpression of SNX16 in SW480 cells significantly promoted tumor growth by 122% and increased the tumor weight by 103% compared to the negative controls (Fig. 6A). Immunohistochemical analysis showed that the SNX16 expression levels were positively correlated with the levels of eEF1A2, c‐Myc, and Ki67 (Fig. 6B). These findings were further validated by western blot analyses of these tumor specimens (Fig. 6C).

### 3.9 SNX16 is positively related to eEF1A2 and c‐Myc expression in CRC patient samples

**Figure 7:**
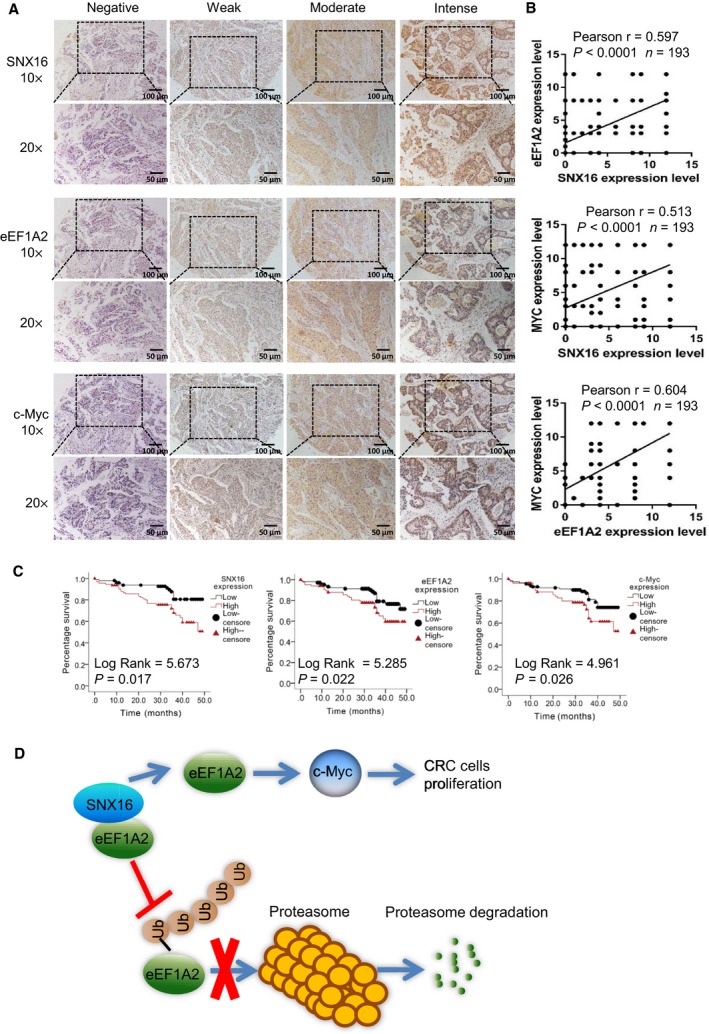
SNX16 expression is positively correlated with the eEF1A2 and c‐Myc levels in CRC patient samples. (A) IHC analysis of SNX16, eEF1A2, and c‐Myc in primary human CRC tissues. Scale bar, 100 μm (10×), 50 μm (20×). (B) Regression analysis showed a positive correlation among SNX16, eEF1A2, and c‐Myc expression in primary CRC (*n* = 193). (C) Overall survival of CRC patients was analyzed using the Kaplan–Meier method. Survival analysis was performed according to the expression of SNX16, eEF1A2, and c‐Myc. Increased expression levels of SNX16, eEF1A2, and c‐Myc were related to the poor patient outcome. (D) Schematic of the regulatory mechanisms of SNX16 in CRC. SNX16 binds to eEF1A2 to stabilize it by inhibiting its ubiquitin‐mediated degradation, which subsequently activates the c‐Myc signaling pathway to mediate the oncogenic effect.

We evaluated the correlations among SNX16, eEF1A2, and c‐Myc expression in a human CRC TMA (*n* = 193) by IHC. Spearman's correlation analyses revealed positive relationships among SNX16, eEF1A2 (*r* = 0.597; *P* < 0.0001), and c‐Myc (*r* = 0.513; *P* < 0.0001) expression, and a positive relationship between eEF1A2 and c‐Myc (*r* = 0.604; *P* < 0.0001) expression in 193 CRC specimens (Fig. 7A,B). Kaplan–Meier analysis showed that increased levels of SNX16, eEF1A2, and c‐Myc expression were correlated with poor overall survival (Fig. 7C). Furthermore, multivariable Cox regression analysis showed SNX16, eEF1A2, and c‐Myc expression levels were independent prognostic factors for poor survival (Table S1).

## 4 Discussion

Sorting nexins are a diverse group of proteins that contain the SNX‐PX domain and play a key role in membrane trafficking (Teasdale and Collins, 2012; Worby and Dixon, 2002). Earlier studies have shown that SNXs are involved in the regulation of important signaling pathways associated with cancers, such as EGFR signaling (Kurten *et al*., 1996; Nishimura *et al*., 2012), Wnt signaling (Sun *et al.*, 2016), TGF‐β signaling (Hao *et al.*, 2011), and so on. SNX16 is a unique SNXs family protein, consisting of a central PX domain, a potential coiled‐coil domain, and a C‐terminal region. The PX domain of SNX16 specifically binds to PI3P, which is mainly distributed in early endosomes and recycling endosomes, whereas the CC domain participates in the homo‐dimerization of SNX16 (Choi *et al.*, 2004; Hanson and Hong, 2003; Le Blanc *et al.*, 2005). SNX16 has been implicated in the development of various tumors. It is reported that SNX16 was overexpressed in the blood cells of bladder cancer patients (Osman, 2006) and ovarian cancer tissues (Pharoah *et al.*, 2013). Previous studies have suggested that SNX16 exhibited alternative splicing in certain melanoma cell lines and could interact with 32 SNPs, which is a known risk factor for prostate cancer (Tao *et al.*, 2012; Watahiki *et al.*, 2004). However, SNX16 was also reported to stimulate EGF receptor degradation in COS‐7 cells (Choi *et al.*, 2004) and a previous study has shown that SNX16 overexpression in MCF‐7 breast cancer cell lines could decrease migration and tumor size in a mouse xenograft model (Zhang *et al.*, 2013). In addition, Xu et al claimed that SNX16 regulated the recycling trafficking of E‐cadherin and inhibited epithelial–mesenchymal transition in renal cell carcinoma (Xu *et al.*, 2017). Nevertheless, a recent study has also shown that E‐cadherin can limit reactive oxygen species‐mediated apoptosis and thereby enhance tumor cell proliferation and survival (Padmanaban *et al.*, 2019). Hence, although SNX16 was related to cancers in various aspects, the precise expression pattern and functional roles of SNX16 in tumors remain exclusive. In this regard, we conducted a more detailed investigation of the expression, prognostic value, functional roles, and underlying mechanism of SNX16 in CRC.

In public datasets analysis, we found a significant increase of SNX16 mRNA level in primary colorectal tumors as compared to their normal mucosa. Consistently, data from our qRT‐PCR, western blot, and IHC analysis displayed the substantial upregulation of SNX16 in CRC tissues. What's more, Kaplan–Meier survival analysis suggested that high SNX16 level was positively associated with poor outcomes of CRC patients, especially in stage III or IV patients with CRC. Univariate Cox regression analysis showed high SNX16 expression was associated with an increased risk of death (HR: 1.64, 95% CI: 1.079–2.500; *P* = 0.021). In particular, multivariate Cox regression analysis also showed high SNX16 expression was an independent risk factor for poorer overall survival after adjustment for risk factors including differentiation and AJCC stage (HR: 1.75, 95% CI: 1.113–2.737; *P* = 0.015) in patients with CRC.

On functional verification, we performed a series of *in vitro* and *in vivo* experiments. Our results showed that ectopic expression of SNX16 in CRC cells significantly increased cell proliferation, repressed apoptosis under stressed conditions, and reduced cell cycle arrest, while reverse regulations were seen in cells with SNX16 knockdown. In keeping with *in vitro* results, subcutaneous xenograft mice model demonstrated that knockdown of SNX16 could significantly inhibit tumorigenesis, whereas ectopic expression of SNX16 significantly promoted tumorigenesis. Our gain of function and loss of function studied both *in vitro* and *in vivo* clearly demonstrated a carcinogenic role of SNX16 in CRC.

Since the underlying molecular mechanisms of SNX16 in CRC remain poorly identified, in our work, we conducted GSEA analysis in four GEO datasets to explore the signaling pathways related to SNX16. Using intersection analysis, we found that only MYC signature was significantly enriched in the SNX16‐overexpressing CRC group in all four databases involved. The proto‐oncogene c‐Myc can regulate multiple genes *via* both transcriptional amplification and co‐factor‐dependent activation/repression. c‐Myc thus drives numerous biological pathways including cell proliferation (Luo *et al.*, 2016), cell cycle (Zhang *et al.*, 2019), metabolism (Fang *et al.*, 2019; Shen *et al.*, 2019a), and apoptosis (Sheikh Zeineddini *et al.*, 2019). c‐Myc expression is strictly controlled at multiple levels, including transcription, stability of both mRNA and protein, and translation (Jiang *et al.*, 2013). As aberrantly high expression of c‐Myc is a common basis of colorectal tumorigenesis (Gong *et al.*, 2018), so we further speculated whether c‐Myc was a functional downstream of SNX16 in CRC cells. Results showed overexpression of SNX16 markedly increased c‐Myc expression, while knockdown of SNX16 expression significantly reduced c‐Myc expression. To further verify whether c‐Myc was involved in the regulation of SNX16‐mediated proliferation of CRC cells, we treated CRC cells with a c‐Myc inhibitor 10058‐F4 to block the expression of c‐Myc. Our results revealed that cells with SNX16 overexpression were much more sensitive to 10058‐F4 treatment and inhibition of c‐Myc could significantly block SNX16 overexpression‐mediated proliferation of CRC cells. In the meantime, the tumor‐suppressive functions of SNX16 knockdown were significantly reversed by c‐Myc overexpression. Collectively, these data implied that the SNX16 might drive CRC cell proliferation *via* regulating c‐Myc signaling.

As we failed to identify the interaction between SNX16 and c‐Myc, we further screened the interacting proteins of SNX16 by using immunoprecipitation–mass spectrometry. Results showed that eEF1A2 might be a novel interaction partner of SNX16. It is reported that eEF1A2 is an important protein involved in protein translation elongation, and eEF1A2 was identified as a putative oncogene in many human cancers (breast, ovary, liver, pancreas, lung, and prostate) (Giudici *et al.*, 2019; Lee *et al.*, 2013; Liu *et al.*, 2019; Pellegrino *et al.*, 2014; Worst *et al.*, 2017; Zang *et al.*, 2015). Researches have indicated that eEF1A2 played important roles in the regulation of various biological processes of cells, such as cell cycle, cell apoptosis, and cytoskeletal regulation. In addition, knockdown of eEF1A2 could cause G1 or G2/M arrest in the cell cycle of cancer cells according to Lee *et al'*s (2013) work. In hepatocellular carcinoma, Pellegrino *et al*. claimed that eEF1A2 protein could promote cell cycle progression (Pellegrino *et al.*, 2014). Therefore, we suspected that eEF1A2 might be an important and functional downstream of SNX16 in CRC cells.

In our work, we found that eEF1A2 protein level upregulated following SNX16 overexpression and downregulated after SNX16 knockdown, while the mRNA level did not show consistent changes. As previous research suggested that eEF1A2 could be degraded *via* the ubiquitin–proteasome pathway (Sanges *et al.*, 2012), so we wondered SNX16 might regulate eEF1A2 expression *via* regulation of ubiquitination. Using MG132 treatment, CHX treatment, and K48‐linked ubiquitination detection, we identified that SNX16 could inhibit the proteasome‐dependent ubiquitination of eEF1A2 protein. To further explore whether eEF1A2 is the key regulator between SNX16 and c‐Myc, we constructed stable SNX16‐overexpressed and eEF1A2‐knockdown SW480 cells and detected c‐Myc expression. Our results indicated that knockdown of eEF1A2 reversed SNX16 induced increased expression of c‐Myc. On the functional rescue, results showed that knockdown of eEF1A2 could reverse the proliferation promoted effect driven by SNX16. Therefore, we concluded that SNX16/eEF1A2/c‐Myc might be a novel regulatory axis that drives tumorigenesis in CRC.

Our study has a few disadvantages. In this study, we found the mechanism that SNX16 activates the c‐Myc signaling pathway by regulating eEF1A2 expression. Nevertheless, the mechanism by which eEF1A2 regulates c‐Myc signaling still remains unclear. Thus, more experiments are needed to fully elucidate the mechanism underlying eEF1A2‐mediated c‐Myc activation. Moreover, our work revealed that SNX16 overexpression led to decreased ubiquitination of eEF1A2 and stabilized eEF1A2 by inhibiting the ubiquitin‐mediated degradation of this protein. However, the process by which SNX16 antagonizes substrate ubiquitination has not been completely understood. Therefore, further experiments are needed to elucidate the mechanism underlying the role of SNX16 in the ubiquitination of eEF1A2.

## 5 Conclusion

In summary, our work firstly reveals that SNX16 is overexpressed in CRC tissues and is closely associated with poor survival of CRC patients. SNX16 plays a critical role in regulating the expression of eEF1A2, then activated c‐Myc singling pathway, which is responsible for the proliferation of CRC cells (Fig. 7C). Our study indicates that SNX16 may be a promising therapeutic target for the inhibition of uncontrolled CRC cell growth *via* the c‐Myc signaling pathway.

## Conflict of interests

The authors declare no conflict of interest.

## Author contributions

HD, ZS, YF, GL, and YL were responsible for the concept and experimental design. YL and XF carried out the experiments. ML, YW, and TM contributed to clinical sample collection. YZ and XL contributed to scoring of immunohistochemical sections. ZL and ML helped with the animal study. YL performed statistical analysis. YL, YF, ZS, HD, and JW were involved in drafting and revision of the manuscript. All authors read and approved the final manuscript.

## Ethics approval and consent to participate

This study was approved by the Institutional Research Medical Ethics Committee of Nanfang Hospital. All animal studies were performed with approval from the Institutional Animal Care and Use Committee of Nanfang Hospital.

## Supporting information


**Fig. S1.** Expression of SNX16 in data from the Onconmine database.
**Fig. S2.** Correlation of SNX16 expression with patient survival in CRC.
**Fig. S3.** qRT‐PCR and Western blot analyses of SNX16 expression in CRC cell lines.
**Fig. S4.** Images of EdU assay in indicated cell lines, related to Fig. 2D. Scale bar, 50 μm.
**Fig. S5.** Knockdown or overexpression of SNX16 did not affect CRC cells migration.
**Fig. S6.** Cell cycle analysis. Cells were enriched in G1 phase by incubation with 0.8 mm l‐mimosine for 24 h.
**Fig. S7.** Cell survival assays were used to determine the vulnerability of cells (LV‐NC vs. LV‐SNX16) to 10058‐F4 treatment.
**Fig. S8.** Co‐IP was performed to detect the interaction between SNX16 and c‐Myc in HT29 cells.
**Fig. S9.** Knockdown of eEF1A2 inhibited CRC cells proliferation *in vitro*.

**Table S1.** Univariable and multivariable Cox regression analyses of the association of clinical characteristics with the prognosis of 193 CRC patients.
**Table S2.** Antibodies used for Western blotting, coimmunoprecipitation and immunofluorescence.
**Table S3.** Nucleotide sequences of the primers used in this study.
