# Supplementary material for: SNX16 activates c‐Myc signaling by inhibiting ubiquitin‐mediated proteasomal degradation of eEF1A2 in colorectal cancer development
Source: Mol Oncol. 2020 Jan 10;14(2):387–406. doi: 10.1002/1878-0261.12626 (PMC6998659; doi:10.1002/1878-0261.12626)
Supplement: Supplementary file 1 — Fig. S1. Expression of SNX16 in data from the Onconmine database. Fig. S2. Correlation of SNX16 expression with patient survival in CRC. Fig. S3. qRT‐PCR and Western blot analyses of SNX16 expression in CRC cell lines. Fig. S4. Images of EdU assay in indicated cell lines, related to Fig. 2D. Scale bar, 50 μm. Fig. S5. Knockdown or overexpression of SNX16 did not affect CRC cells migration. Fig. S6. Cell cycle analysis. Cells were enriched in G1 phase by incubation with 0.8 mm l‐mimosine for 24 h. Fig. S7. Cell survival assays were used to determine the vulnerability of cells (LV‐NC vs. LV‐SNX16) to 10058‐F4 treatment. Fig. S8. Co‐IP was performed to detect the interaction between SNX16 and c‐Myc in HT29 cells. Fig. S9. Knockdown of eEF1A2 inhibited CRC cells proliferation in vitro. [file MOL2-14-387-s001.pdf]

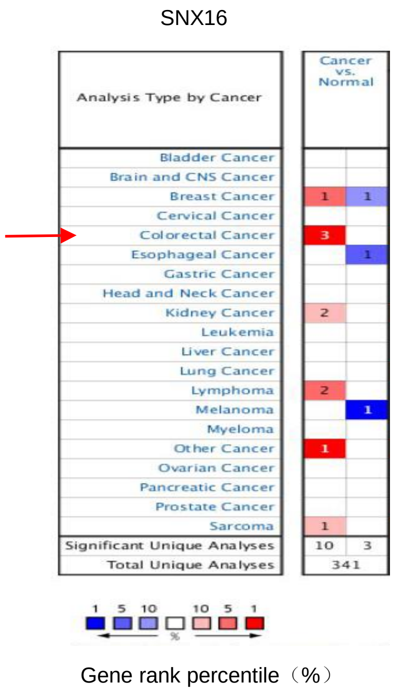

Figure S1. Expression of SNX16 in data from the Onconmine database. Analysis of SNX16 expression in various types of malignancies. Red indicates upregulation, and blue indicates downregulation.

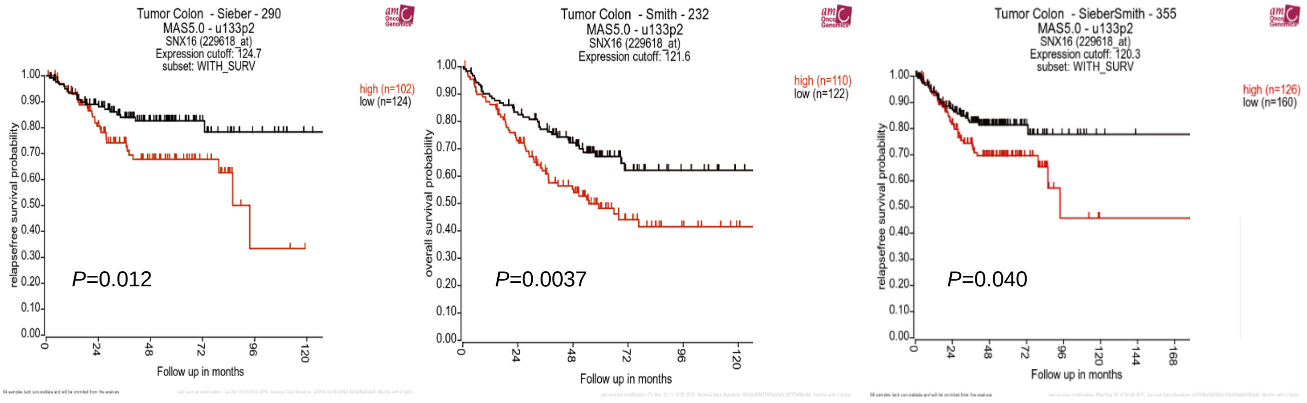

Figure S2. Correlation of SNX16 expression with patient survival in CRC. Probabilities of overall survival or relapsefree survival were obtained from the R2: Genomics Analysis and Visualization Platform (http://r2.amc.nl).

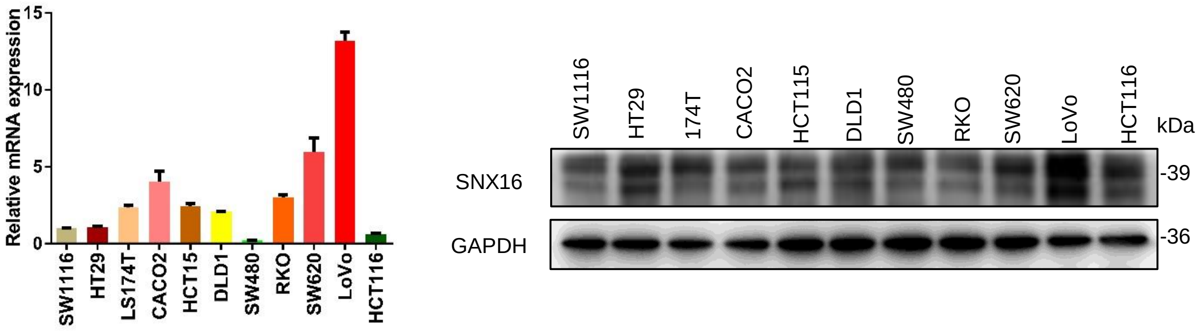

Figure S3. qRT-PCR and Western blot analyses of SNX16 expression in CRC cell lines.

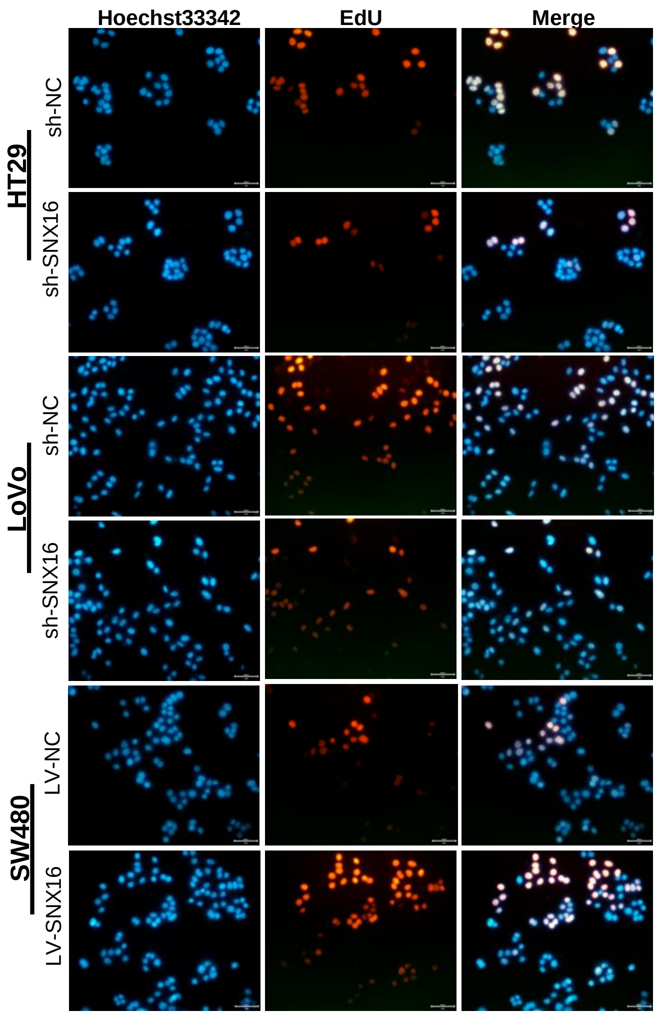

Figure S4. Images of EdU assays in indicated cell lines, related to Fig 2d. Scale bar, 50 μm.

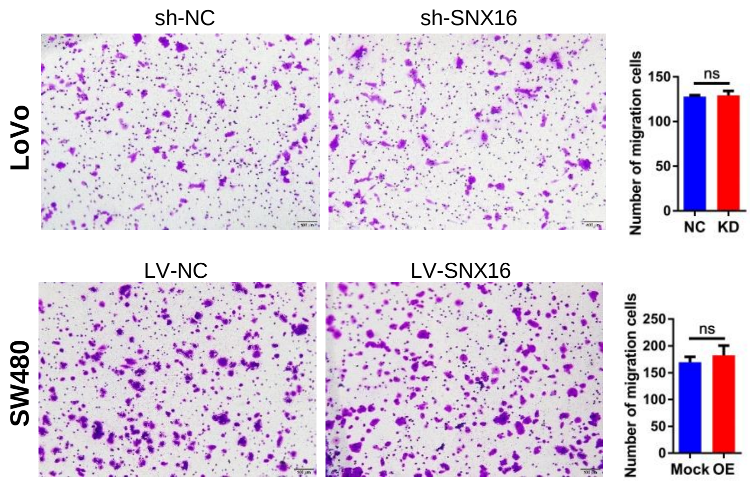

Figure S5. Knockdown or overexpression of SNX16 did not affect CRC cells migration. The results are shown as the means ± SEMs (n=3); ns=non-significant (P>0.05).

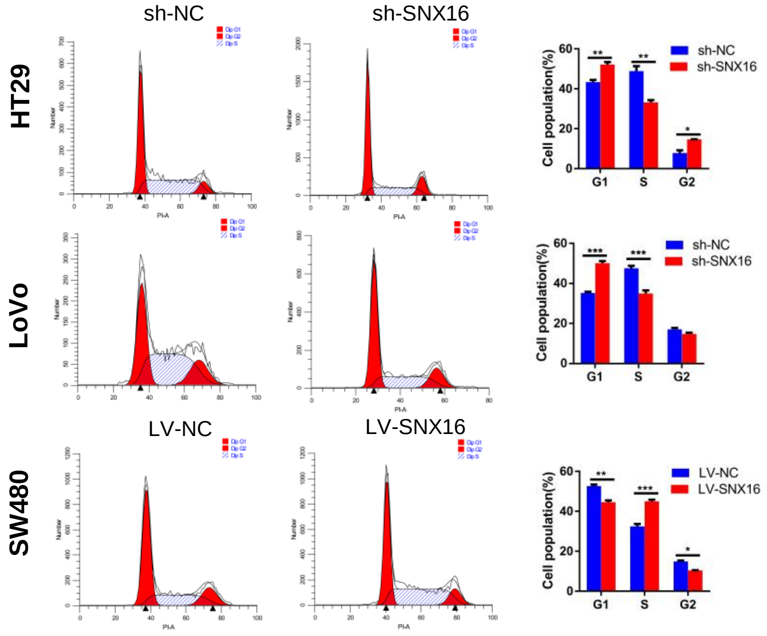

Figure S6. Cell cycle analysis. Cells were enriched in G1 phase by incubation with 0.8 mM L-minosine for 20 hour. Cells were released and collected for flow cytometry analysis at the indicated time points. **P*<0.05 ,***P*<0.01, ****P*<0.001.

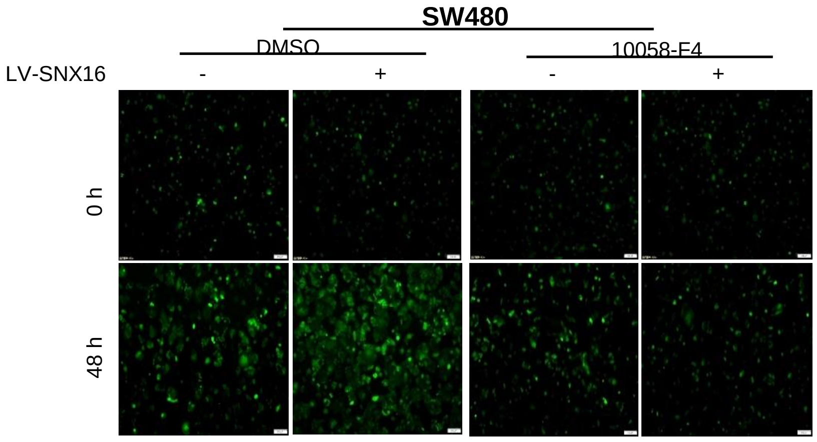

Figure S7. Cell survival assays were used to determine the vulnerability of cells (LV-NC vs. LV-SNX16) to 10058-F4 treatment. Scale bar, 100 µm.

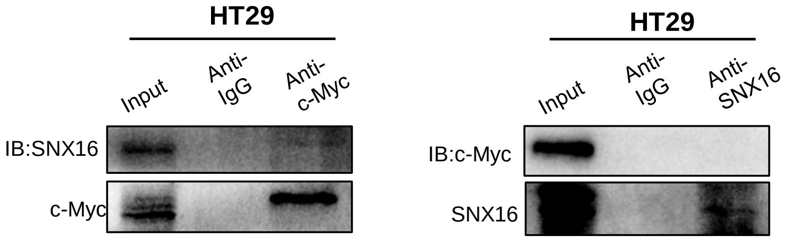

Figure S8. Co-IP was performed to detect the interaction between SNX16 and c-Myc in HT29 cells.

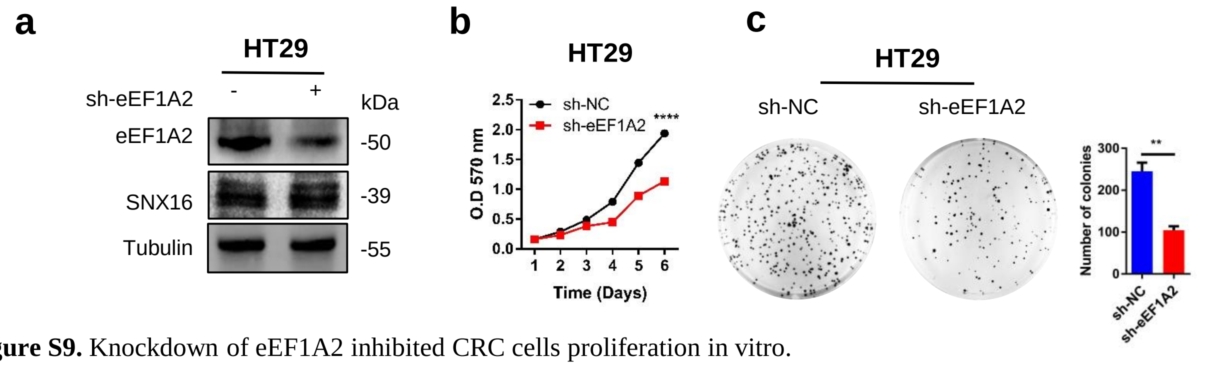

Figure S9. Knockdown of eEF1A2 inhibited CRC cells proliferation in vitro.

a. The expression of SNX16 and eEF1A2 in eEF1A2-knockdown cells were measured by Western blotting. Tubulin was used as the loading control.

b. MTT assay. The results are shown as the means ± SEMs (n=5); ****P<0.0001.

c. Colony formation assays. The results are shown as the means ± SEMs (n=3); **P<0.01.
