# Supplementary material for: SNX16 activates c‐Myc signaling by inhibiting ubiquitin‐mediated proteasomal degradation of eEF1A2 in colorectal cancer development
Source: Mol Oncol. 2020 Jan 10;14(2):387–406. doi: 10.1002/1878-0261.12626 (PMC6998659; doi:10.1002/1878-0261.12626)
Supplement: Supplementary file 2 — Table S1. Univariable and multivariable Cox regression analyses of the association of clinical characteristics with the prognosis of 193 CRC patients. Table S2. Antibodies used for Western blotting, coimmunoprecipitation and immunofluorescence. Table S3. Nucleotide sequences of the primers used in this study. [file MOL2-14-387-s002.docx]

**Supplementary tables**

**Table S1. Univariable and multivariable Cox regression analyses of the association of clinical characteristics with the prognosis of 193 CRC patients.**

| **Variables** | **HR (95% CI)** | ***P*-value** | |
| --- | --- | --- | --- |
| **Univariable Analysis^1^** |  | |  |
| Gender （male vs female） | 1.185(0.646-2.174) | | 0.583 |
| Age (years)（<65 vs ≥65） | 1.470(0.792-2.729) | | 0.222 |
| Differentiation (W/M vs P)^2^ | 2.074(1.022-4.210) | | 0.043 |
| TNM stage (I/II vs III/IV) | 2.900(1.548-5.432) | | 0.001 |
| SNX16 expression (low vs high) | 2.075(1.118-3.854) | | 0.021 |
| eEF1A2 expression (low vs high) | 2.002(1.090-3.680) | | 0.025 |
| c-Myc expression (low vs high) | 1.948(1.067-3.557) | | 0.030 |
| **Multivariate Analysis I^1^** |  | |  |
| Differentiation (W/M vs P)^2^ | 1.834(0.889-3.781) | | 0.100 |
| TNM stage (I/II vs III/IV) | 2.717(1.436-5.139) | | 0.002 |
| SNX16 expression (low vs high) | 2.204(1.180-4.116) | | 0.013 |
| **Multivariate Analysis II^1^** |  | |  |
| Differentiation (W/M vs P)^2^ | 1.794(0.873-3.685) | | 0.112 |
| TNM stage (I/II vs III/IV) | 2.621(1.388-4.948) | | 0.003 |
| eEF1A2 expression (low vs high) | 1.977(1.071-3.649) | | 0.029 |
| **Multivariate Analysis III^1^** |  | |  |
| Differentiation (W/M vs P)^2^ | 1.613(0.783-3.316) | | 0.195 |
| TNM stage (I/II vs III/IV) | 2.822(1.487-5.355) | | 0.002 |
| c-Myc expression (low vs high) | 2.048(1.116-3.756) | | 0.021 |

HR hazard ratio, CI confidence interval

^1^Cox regression model (method = Enter)

^2^W, well; M, moderate; P, poor

**Table S2. Antibodies used for Western blotting, coimmunoprecipitation and immunofluorescence.**

| Antibody | Manufacturer | Country | dilution |
| --- | --- | --- | --- |
| Western blotting | | |  |
| SNX16 | Abcam | USA | 1:500 |
| Anti-Ubiquitin (linkage-specific K48) | Abcam | USA | 1:500 |
| p21 | Cell Signalling Technology | USA | 1:1000 |
| p18 | Cell Signalling Technology | USA | 1:1000 |
| CDK6 | Cell Signalling Technology | USA | 1:1000 |
| Cyclin D1 | Cell Signalling Technology | USA | 1:1000 |
| Cyclin D3 | Cell Signalling Technology | USA | 1:1000 |
| eEF1A2 | Proteintech | USA | 1:500 |
| c-Myc | Proteintech | USA | 1:500 |
| Caspase 3 | Proteintech | USA | 1:500 |
| Cleaved caspase 3 | Proteintech | USA | 1:500 |
| Tubulin | Proteintech | USA | 1:500 |
| GAPDH | Proteintech | USA | 1:500 |
| Coimmunoprecipitation | | |  |
| SNX16 | Santa Cruz | USA | 1:50 |
| eEF1A2 | Proteintech | USA | 1:50 |
| Immunofluorescence | | |  |
| SNX16 | Santa Cruz | USA | 1:100 |
| eEF1A2 | Proteintech | USA | 1:200 |

**Table S3. Nucleotide sequences of the primers used in this study**

| **Gene** | **Forward primer** | **Reverse primer** |
| --- | --- | --- |
| SNX16 | TGCACTTGAGGTTGATCAAGATGTC | CAGCATCATATGCCACTTCTGCTAC |
| eEF1A2 | CCATGTGTGTGGAGAGCTTCTC | TCTCCACGTTCTTGATGACGCC |
| GAPDH | GCACCGTCAAGGCTGAGAAC | TGGTGAAGACGCCAGTGGA |
